# Coho Salmon (*Oncorhynchus kisutch*) Prefer and Are Less Aggressive in Darker Environments

**DOI:** 10.1371/journal.pone.0151325

**Published:** 2016-03-30

**Authors:** Leigh P. Gaffney, Becca Franks, Daniel M. Weary, Marina A. G. von Keyserlingk

**Affiliations:** Animal Welfare Program, Faculty of Land and Food Systems, University of British Columbia, 2357 Main Mall, Vancouver, BC, V6T 1Z6, Canada; Swansea University, UNITED KINGDOM

## Abstract

Fish are capable of excellent vision and can be profoundly influenced by the visual properties of their environment. Ambient colours have been found to affect growth, survival, aggression and reproduction, but the effect of background darkness (i.e., the darkness vs. lightness of the background) on preference and aggression has not been evaluated systematically. One-hundred Coho salmon (*Oncorhynchus kisutch*), a species that is increasing in popularity in aquaculture, were randomly assigned to 10 tanks. Using a Latin-square design, every tank was bisected to allow fish in each tank to choose between all the following colour choices (8 choices in total): black *vs*. white, light grey, dark grey, and a mixed dark grey/black pattern, as well as industry-standard blue *vs*. white, light grey, dark grey, and black. Fish showed a strong preference for black backgrounds over all other options (p < 0.01). Across tests, preference strength increased with background darkness (p < 0.0001). Moreover, having darker backgrounds in the environment resulted in less aggressive behaviour throughout the tank (p < 0.0001). These results provide the first evidence that darker tanks are preferred by and decrease aggression in salmonids, which points to the welfare benefits of housing farmed salmon in enclosures containing dark backgrounds.

## Introduction

In general, fish rely on vision to detect prey, predators, and mates [1–3] and exhibit one of the greatest visual sensitivities among vertebrates [4]. A large body of literature has shown that fish are strongly affected by the visual properties of their environment: light conditions, substrate colour, and tank wall colour can impact fish stress responses, growth, and aggression [5–10]. Across these studies, there is some evidence that darkness—how much light the environment absorbs rather than reflects [11]—may be a critical dimension. For example, Nile tilapia (*Oreochromis niloticus*) and summer flounder (*Paralichthys dentatus*) show lower plasma cortisol levels in darker versus lighter environments [9]. Höglund et al. [12], found that pairs of Arctic charr (*Salvelinus alpinus*) interacting on a black background displayed darker colouration and less aggression than fish interacting on a white background. Collectively, these studies suggest that fish may prefer dark environments and that having access to dark environments may also reduce aggression.

With the demand for fish projected to increase by almost 50 million tonnes and aquaculture supplying approximately 73% of this growth [13], there is increasing interest in developing effective and humane land based captive rearing systems. Surprisingly little attention has been paid to the environmental parameters that would best promote fish welfare. Along with the ethical implications, improved fish welfare has the potential to improve industry production quality and quantity as well as public perception and product acceptance [14–16]. Previous research on the importance of the visual properties of the environment suggests that even simple choices such as tank colour could dramatically affect fish health [9,10,12]. Though tanks could easily be fabricated in any colour, the prevailing industry practice has become to house farmed fish in light blue tanks.

The aim of this study was to assess tank colour preferences of Coho salmon (*Oncorhynchus kisutch)* and quantify how background colour affects aggression. Extending the previous research, we focused specifically on background darkness—testing several shades of gray including black and white—against the industry standard light blue. As an initial exploration into the possibility that fish might prefer certain backgrounds because they allow for better concealment, we also tested a black background (absolute darkness) against a dark, dappled background that was lighter but thought to provide better camouflaging properties.

## Methods

### Ethics Statement

This protocol was approved by the University of British Columbia Animal Care Committee (application number: A13-0210). All experimental procedures were performed in accordance with the Canadian Council on Animal Care guidelines on care and use of fish in research.

### Animals and Housing

One hundred, 2-year-old coho salmon (mixed sex, mean ± s.d. weight 100 ± 5 g) were obtained from a collaborating research laboratory, originally purchased from Target Marine Hatcheries Ltd. (Sechelt, BC, Canada). Fish were reared during this time in circular, 244 cm diameter (5.6m^3^), light blue, fibreglass tanks (D&T Fiberglass Inc., Sacramento, CA, USA).

Fish were randomly assigned to groups of ten, each housed in a circular 600 L, 107 cm diameter (0.7m^3^), light blue, fibreglass tank (D&T Fiberglass Inc., Sacramento, CA, USA). The 10 tanks were serviced by a freshwater recirculating system providing mechanical and biological filtration, UV-sterilization, and oxygen injection (components were supplied by Integrated Aqua Systems, Inc., Escondido, CA, USA). During the experiment, mortality, water temperature, dissolved oxygen content, and pH were measured daily and ammonia-N was measured weekly. No mortalities occurred. Water temperature was maintained between 8 and 12°C. The dissolved oxygen content of the water was kept between 70–90% air saturation, pH between 6.0 and 6.8 and ammonia-N was less than 0.5 ppt. The average water flow rate was 360 L/min (± 1 L/min). These water quality parameters are well within the range of what salmon would experience in the wild [17] and adhere to industry standard ranges [18]. Tanks were cleaned once a week.

Fish were hand fed to satiety with a commercially available pelleted diet (Transfer Plus 3.0MM and 4.0MM, Skretting Canada, Vancouver, BC, Canada) once daily from Monday to Saturday at 1000h; no food was given on Sunday. All experimental populations were housed under a 10-h light; 14-h dark cycle (lights on and off at 0730h and 1700h, respectively). To ensure even light distribution in each tank and to avoid complications due to room lighting, a full spectrum daylight fluorescent lamp (CFL T2 23W Daylight Bulb, Globe Electric, Montreal, QC, Canada) was suspended 1 m above the center of each tank and light intensity was adjusted to 410 lx at water surface.

### Experimental Procedures

The interior sides and base of each of the experimental tanks were lined with laminated paper (InPrint Graphics & Copying Ltd., Vancouver, BC, Canada) varying in pigmentation: black (0°, 0%, 0%; hue, saturation, value, respectively), white (0°, 0%, 100%), light grey (0°, 0%, 85%), dark grey (0°, 0%, 45%), and a mixed dark grey/black dappled/sponge pattern. The industry-standard, original blue tank background had a hue of 200.4°, a saturation of 22.8%, and a value of 85% (the same % value as the light grey laminated sheets). Sixteen 1.27cm x 0.32cm magnets (Rare-Earth Circular Magnets, Lee Valley Tools Ltd. and Veritas Tools Inc., Vancouver, BC, Canada) were used to hold the laminated pieces of paper in place in each tank. To habituate fish to each of the conditions, tanks were lined with each background option for a total of 3 days before preference testing began. After each individual preference test, the tanks were lined with the subsequent preference test background options and preference testing began 3 days afterwards.

Preference tests were conducted between (a) black versus white, light grey, dark grey and patterned and (b) blue versus white, light grey, dark grey, and black. Every tank experienced every preference test. To avoid order effects, exposure was set by a replicated 5x5 Latin square such that on any given day, every tank experienced a different colour choice option in a randomized order. All tanks were split into two to provide fish a choice of background colour. To avoid an effect of side on preferences, colour placement was alternated across the 10 tanks.

The positions of the fish in each tank were video recorded from above using a Microsoft LifeCam StudioTM webcam (USA). Experimental trials began 15 min after the camera was placed above the tank to control for disturbances. The fish in each tank were recorded for a total of 10 min, starting at 1100h. Videos were scored using scan sampling at 30-s intervals to determine the number of fish on each side of the tank, yielding 20 observations/trial.

Videos were scored continuously for aggressive acts (charging, a rapid dart towards another fish with attempts to bite the other fish, and chasing, rapid pursuit of another fish) throughout each 10-min trial.

### Statistical Methods

To determine background darkness preference, we ran one-sample t-tests on the average number of fish per side using 5 (half the number of fish per tank) as the critical value. Across trials, we modelled the effect of background darkness on preference strength (the degree to which an animal prefers a choice in a preference test) using a multilevel model with fixed effects of background darkness (continuous from 0 [white] to 1 [black]), blue (coded as an indicator/dummy variable of black vs. blue trial) and pattern (indicator variable of a pattern vs. regular trial) and a random effect of tank to control for repeated testing of tanks and to avoid pseudoreplication [19–21].

To model aggression on each side of the tank, we log-transformed aggressive-behaviour-per-fish (side-aggression = log[aggressive acts per side/average number of fish per side across the trial]) as a function of side-darkness (continuous) and blue and pattern indicator variables with a multilevel model that included tank and trial as nested random effects. For total number of aggressive acts per trial, we applied generalized multilevel models (Poisson error structure and log link) with a random effect of tank and fixed effects for mean tank darkness (the average darkness of the two sides), blue and pattern indicator variables, and whether or not the trial contained a side with greater than 0.55 darkness (a refuge).

For all data processing, modeling, and plotting, we used R v. 3.2.0 [22] and the following packages: plyr [23], reshape [24], lme4 [25], and lmerTest [26].

## Results

### Tank Colour Preference

**Fig 1:**
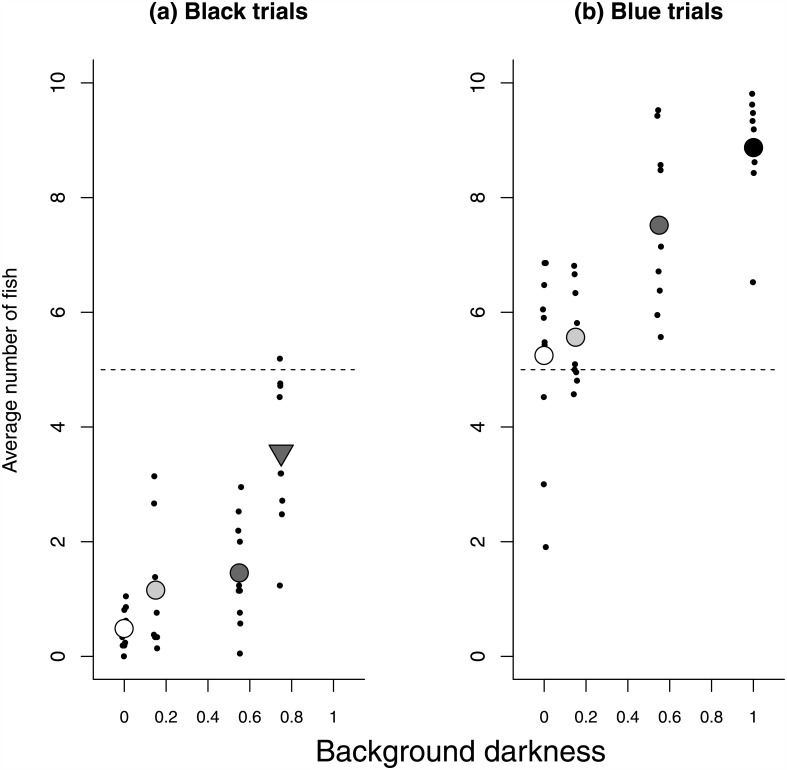
Salmon prefer darker backgrounds. Preference strength increased with increasing background darkness (p < 0.0001). For the (a) black-trials and (b) blue-trials, dots represent each tank’s average number of Coho salmon (*Oncorhynchus kisutch*) located on the comparison side. Background darkness ranges from 0 (white) to 1 (black). Large symbols represent average values across all tanks (n = 10) with the fill colour corresponding to the background darkness of the comparison side (white, light grey, dark grey, or black) and the triangle (▿) indicating the patterned background trial.

Pair-wise comparisons for the black-trials revealed a strong preference for black background over all other options (black vs. white: t(9) = 41.24, p < .0001; black vs. light grey: t(9) = 11.88, p < 0.0001; black vs. dark grey: t(9) = 12.11, p < 0.0001; black vs. pattern: t(9) = 3.66, p < .01; Fig 1a). In the blue-trials, fish showed no preference for white vs. blue (t(9) = 0.47, p > 0.6), a tendency to prefer light grey over blue (t(9) = 2.22, p < 0.1), and a preference for both dark grey and black over blue (t(9) = 5.63, p < 0.001, and t(9) = 13.06, p < 0.0001, respectively; Fig 1b). Across all trials, we found that preference strength increased with background darkness (t(67) = 8.81, p < 0.0001, Fig 1a and 1b). We found limited evidence that the patterned background was preferred beyond what would be expected based on its darkness value alone (t(67) = 1.92, p = 0.06, Fig 1b).

### Aggressive Acts

**Fig 2:**
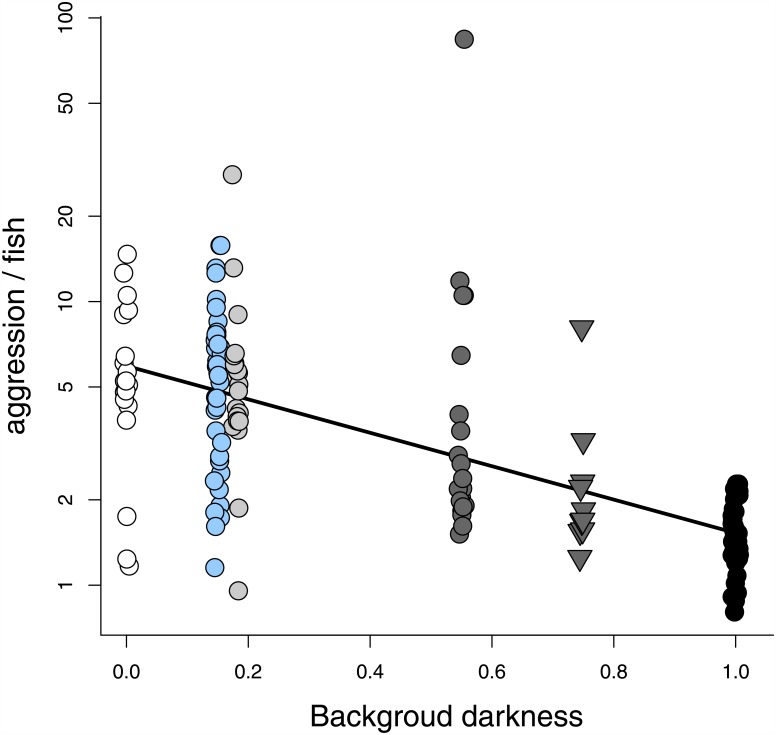
Dark backgrounds decrease aggression. Average aggression (the total number of aggressive acts displayed divided by the average number of fish across a 10-min trial) on a side (log scale) decreased with background darkness (p < 0.0001). Background darkness ranges from 0 (white) to 1 (black). Filled-in circle (◯) colour corresponds to the background colour (white, blue, light grey, dark grey, or black) and the triangle (▿) indicates the patterned background.

**Fig 3:**
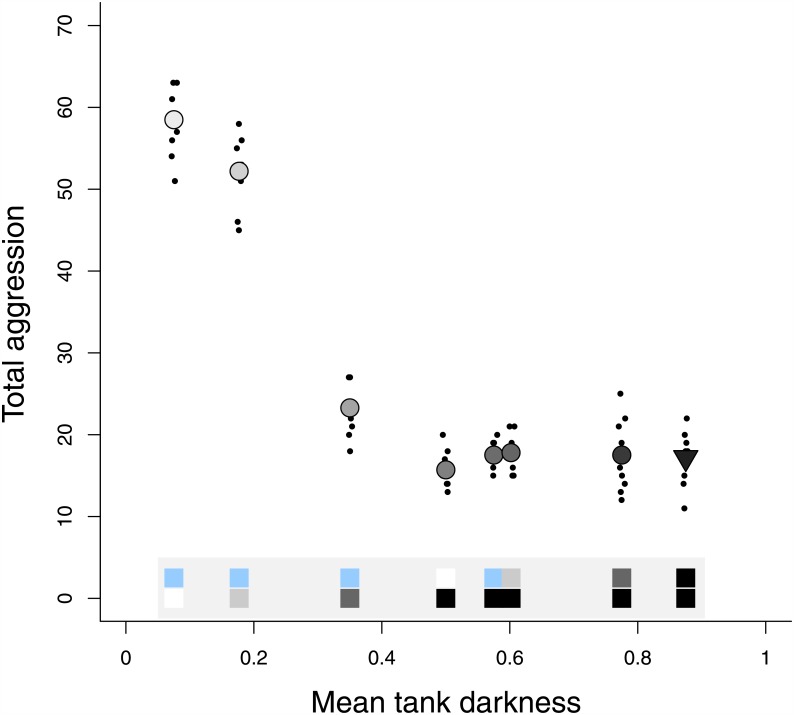
Dark refuges decrease aggression throughout the tank. Overall aggression (total number of aggressive acts occurring per 10-min trial) throughout the tank decreased with average tank darkness (p < 0.01), but especially when the tank contained an area available with darkness of .55 or greater (p < 0.0001). Dots represent aggression counts in a trial for each tank. Background darkness ranges from 0 (white) to 1 (black). Filled-in circle (◯) colour corresponds to the average tank darkness and the triangle (▿) indicates the average tank darkness of the pattern vs. black trial. The bi-colour panel at the bottom of the plot shows the two colours tested in the trial.

Background darkness dramatically decreased aggression (df = 157, t = 11.06, p < 0.0001) Fig 2): aggression was nearly four times greater on blue backgrounds (the industry standard) than on black backgrounds. The effect of blue and patterned background on aggression was entirely explained by their darkness value or, in other words, aggression on the blue side was no different than aggression on the light grey side (which had the same darkness value) and aggression on the patterned side was consistent with what would have been expected given the patterned background’s average darkness value. There was virtually no variation in aggression per tank—the ICC (intraclass correlation coefficient) was 0. Examining aggression at the tank level, we found that increasing the average darkness of the environment decreased the number of overall aggressive acts (z = 2.79, p < 0.01; Fig 3). Interestingly, however, the decrease in aggression with background darkness was not linear even on a log scale. When the tank contained a dark ‘refuge’—a side of the tank with a darkness value of 0.55 or higher—overall aggression was reduced beyond what would be expected by the effect of mean tank darkness alone. Fish engaged in only one third as many aggressive acts in trials with a dark ‘refuge’ compared to trials without a dark ‘refuge’ (Fig 3; z = 9.41, p < 0.0001).

## Discussion

The fish in this study showed a clear preference for black backgrounds over all other colour options and for darker backgrounds in general. Dark backgrounds also substantially decreased aggression, both in terms of the aggression observed on the background side itself and in terms of aggression observed throughout the tank. We found only limited evidence that patterned backgrounds may be preferred beyond what would be expected given the background darkness value alone and found no evidence that the patterned backgrounds uniquely reduced aggression. A wide range of studies have shown that prey animals prefer parts of the habitat that match their own visual appearance to reduce the risk of predator detection (e.g. [27–29]). Similarly to Kjernsmo and Merilaita [30], we speculate that the presence of a predator or the use of a patterned background that more accurately matches the colouration of coho salmon may have resulted in a higher preference for the dark, dappled background used in our study.

With the rapid expansion of fish aquaculture, the welfare of farmed fish has received increasing attention [31]. However, the majority of studies on aquaculture fish welfare have focused on the biological functioning aspect of welfare, addressing issues such as growth rates, flesh quality, injury, disease, and reproductive problems (e.g. [14–16, 31]). However, good animal welfare goes beyond physical health. It also involves the ability of an animal to express natural behaviours and a lack of mental suffering from pain, fear, etc. [32]. The present study focuses on fish preferences and behaviour as indicators of fish welfare. There is a growing body of physiological, behavioural, and anatomical evidence indicating that fish are able to experience pain and fear (e.g. [33–47]). Thus, affective states, revealed through behaviour and potentially impacted by preferences, should be considered when assessing fish welfare in aquaculture systems.

Preference tests allow animals to express their priorities, giving us insight into what may be important to their health and welfare [48]; they are widely used in the welfare literature, for example, to assess flooring temperatures for sows [49], lighting type and intensity for poultry [50, 51], bedding for dairy cows [52], and nesting material for laboratory mice [53]. However, the results of preference tests vary with context [48] including the animal’s previous experiences [54]. Previous research suggests that animals may initially prefer environments they are familiar with (Dawkins, 1977). In the current study, for example, the fish may have shown a preference for blue tanks over other options because they were previously reared in blue tanks. Our results, however, show that the fish had a clear preference for black backgrounds over all other colour options and for darker backgrounds in general. Moreover, animals do not always make choices that are best for their long-term health and welfare [55]. The implications for welfare of preference tests must therefore be interpreted for with care. For example, in the current study the fish clearly preferred the darker sides, but, in absolute terms, were also observed to engage in more aggressive acts on those sides. However, the higher frequency of aggressive interactions observed on the black sides was explained entirely by the greater fish numbers and higher densities on those sides. In fact, accounting for the fish densities revealed the reverse pattern to be the case: darker colours resulted in lower aggression on a per fish basis.

The preference for darker colours may be explained, in part, by the feeding habits of Coho salmon. Salmon rely almost entirely on vision to detect their prey, which are mostly drifting, light-coloured invertebrates [1, 2]. It is likely that these prey are easier to detect against a dark background, thus leading salmon to have a potentially innate preference for darker environments [56]. Additionally, bright environments, especially those without cover, have been shown to elicit a physiological stress response such as increased plasma cortisol levels for fish [57]. Possibly as a form of anti-predator behaviour [58], fish tend to seek dark environments when anxious [59, 60]. Thus, fish preference for darker background may also be related to protective, escape behaviours.

Salmonids are naturally territorial and aggressive behaviours (charging and chasing) are known to increase with increasing density [31, 61, 62]. Aggression is thus of particular concern in closed containment aquaculture systems [63]. In the present study, darker backgrounds were also shown to reduce overall fish aggression levels within the tank, despite the higher fish densities on the darker sides. Salmonids, like other teleost fish, have the ability to adjust the colour of their skin in response to changes in background colour [64, 65]. In salmon, darkening of the skin and eyes signals social subordination, while lightening of the skin signals dominance [65–67]. Thus, a darker fish may represent less of a threat and elicit less aggression than a conspecific displaying paler body colouration. Our results show that the effect of background darkness on aggression extends beyond the immediate environment: having a discrete area of darkness in the environment reduced aggression throughout the entire environment. Follow up studies should examine this result further by directly comparing environments with equivalent levels of average darkness, but varying whether they contain a dark refuge. Determining the visual parameters that minimize aggression could have important implications for the welfare of fish in captivity.

Salmonids are the most produced species of fish in aquaculture [13] and there is growing interest rearing coho salmon in land-based, closed containment aquaculture systems [18, 68]. Aggression is a major concern in closed containment aquaculture and is often the primary cause of fin and skin damage in fish [31, 61, 62]. Fin and skin damage have the potential to cause inflammation and pain, and may lead to infection, reduced survival in salmon, decreased production and decreased product quality [31, 62, 66, 69, 70]. Furthermore, salmon smolts have a long-history of being cultured in hatcheries [71]. Many studies have investigated how rearing salmonids in semi-naturalistic conditions has the potential to improve the post-release survival and performance of hatchery-reared fish. For example, adding structure to the rearing environment decreases stress levels [72] and promotes survival skills [73]. However, no study to date has investigated the effect of tank colour on survival and success of hatchery-reared fish. Thus management practices such as selecting appropriate tank colours have the potential to improve the welfare of many fish. The present research indicates that tank darkness matters to fish and can alter their social behaviour.

### Limitations and Future Research

One limitation of the current study was that the fish were kept under a relatively narrow range of environmental conditions. Preference tests depend on context, and external and internal variables such as, temperature, age of the animal, or time of the year, must be considered [48]. Preferences may also fluctuate in response to changing variables; for example, pigs prefer to rest on straw in the morning when it is cold, but not in the evening when it is warmer [74]. Future research should determine temporal, spatial and environmental influences on tank darkness preferences.

In the case of wild coho salmon, the majority of their first year of life is spent living in freshwater streams, after which they migrate to the ocean, undergoing a metamorphic process called smoltification [17]. Thus, the salinity of water ranges from 0ppt (freshwater) to 35ppt (saltwater) and temperature ranges from 0°C to 25°C during the lifecycle of a wild coho salmon [17]. Considering the visual pigments of coho salmon are maximally sensitive to a specific region of the spectrum and can be altered as fish develop and undergo migration [4, 75, 76], changes in water salinity or temperature may affect how coho salmon react to different tank colours. Thus future research should examine how colour affects aggression and preference in salmon held in differing water salinity and temperature levels.

In addition, fish age may influence the effect that colour has on aggressive behaviour and colour preference in fish. For example, as juvenile coho salmon mature in the wild, individuals acquire their own territories for feeding and begin to engage in intraspecific aggressive behaviours in the form of territory defense and hierarchy establishment [77]. Thus, coho salmon aggression varies with life stage. Although no research to date has looked at how colour interacts with fish aggression at differing ages, it is possible that as salmon grow and their aggression levels change, colour preferences may also change. The results presented in this study should be considered specific to the age of fish examined.

The stocking density of the tanks in this study, measured using the weight of fish per unit volume of the tanks (expressed as kg/m^3^), was approximately 14 kg/m^3^ (compared to approximately 3 kg/m^3^ used in the other studies), which is considerably lower than what is normally used in commercial salmonid aquaculture farms, where stocking densities in excess of 80 kg/m^3^ are common [18]. It is necessary to determine, therefore, how the effects of tank colour may interact with stocking density. One possibility is that tank colour would have an even greater impact on aggression levels in these larger, more densely stocked tanks. Alternatively, at such high stocking densities, the visual properties of the environment may matter less. More research needs to be conducted at higher densities more similar to those found on commercial farms.
